# Work-Family Conflict and Depressive Symptoms Among Chinese Employees: Cross-Level Interaction of Organizational Justice Climate and Family Flexibility

**DOI:** 10.3390/ijerph17196954

**Published:** 2020-09-23

**Authors:** Mingjie Zhou, Jinfeng Zhang, Fugui Li, Chen Chen

**Affiliations:** 1 CAS Key Laboratory of Mental Health, Institute of Psychology, Chinese Academy of Sciences, Beijing 100101, China; zhoumj@psych.ac.cn (M.Z.); lifg@psych.ac.cn (F.L.); 2 Department of Psychology, University of Chinese Academy of Sciences, Beijing 100049, China; 3 School of Public Affairs, Chongqing University, Chongqing 400044, China; 4 Department of Social and Behavioural Sciences, City University of Hong Kong, Hong Kong 999077, China; cchen323-c@my.cityu.edu.hk

**Keywords:** work-family conflict, depressive symptoms, organizational justice climate, family flexibility

## Abstract

This study aims to examine how organizational and family factors protect employees from depressive symptoms induced by work-family conflict. With a cross-sectional design, a total of 2184 Chinese employees from 76 departments completed measures of work-family conflict, organizational justice, family flexibility, and depressive symptoms. The results showed that work-family conflict including work-to-family conflict and family-to-work conflict was positively associated with depressive symptoms. In cross-level analysis, organizational justice climate weakened the adverse effect of work-family conflict on depressive symptoms and the buffering effects of procedural and distributive justice climate in the association between work-family conflict and depressive symptoms depended on family flexibility. Specifically, compared with employees with high family flexibility, procedural and distributive justice climate had a stronger buffering effect for employees with low family flexibility. These results indicate that organization and family could compensate each other to mitigate the effect of work-family conflict on employees’ depressive symptoms. Cultivating justice climate in organization and enhancing family flexibility might be an effective way to reduce employees’ depressive symptoms.

## 1. Introduction

### 1.1. Work-Family Conflict and Depressive Symptoms among Chinese Employees: Cross-Level Interaction of Organizational Justice Climate and Family Flexibility

Work-family conflict is defined as “a form of interrole conflict in which the role pressures from the work and family domains are mutually incompatible in some respect” ([1], p. 77). In China, with women’s entry into the workforce, the traditional gender roles of breadwinning men and homemaking women are changing. This civilian labor trend increases the likelihood that both men and women face simultaneous management of work and family issues across the life span [2]. In addition, companies put forward high demand for employees in this highly competitive business world. Thus, employees are facing problems of how to balance their work and family lives and suffer from work-family conflict, which may result in depressive symptoms. As an important aspect of employee mental health, depressive symptoms reflect the depression severity [3] and have often been studied in the field of occupational health [4]. Construction is a project-driven industry and presents a strong occupational stress because of the considerable dynamism, uncertainty, and a high requirement on product delivery on time, within budget, and with strict standards [5,6]. Employees in this industry face high-intensity work, late working hours, extensive business travels, and great work pressure, and they easily suffer from work-family conflict and depressive symptoms [5,6]. Many construction workers or engineers need to work outside and leave away from home for a long time. In addition, they have to work overtime when encountering urgent tasks. In such a case, their work-family balance may be disrupted, and they may feel depressed due to the lack of time with family members. Moreover, China, as a developing country, is now implementing many engineering construction projects and a large amount of people are working in the engineering construction industry and their occupational health needs more attention. Thus, this research focused on the work-family conflict and depressive symptoms among Chinese employees in the engineering construction industry.

### 1.2. Work-Family Conflict and Depressive Symptoms

Role theory provides a useful framework to understand work-family conflict [2]. This theory proposes that an individual may lack the time and energy to meet obligations of both work and family. Simultaneous occurrence of work and family roles might result in an increased likelihood of role conflict, overload, and other negative outcomes [7]. Considering the directionality, work-family conflict includes two dimensions of work-to-family conflict (WFC) and family-to-work conflict (FWC) [8]. WFC describes the situation in which the demands of one’s job interfere with family-related responsibilities. Conversely, FWC describes the situation in which the demands of one’s family interfere with work-related responsibilities. Previous studies have shown that WFC and FWC are two different constructs [4]. In this study, the term work-family conflict refers to the conflict between work and family, without referring to directionality. The terms WFC and FWC depict the directionality of conflict between work and family.

Studies in Western culture have demonstrated that individuals experienced more depressive symptoms as their WFC or FWC increased [9,10,11]. In China, with the rapid growth of China’s economy and the shift of traditional gender roles at home and work, the work-family conflict level of both male and female employees has increased significantly over the past years [12]. It is necessary to conduct more research on Chinese employees’ work-family conflict and mental health. However, there are only few studies that reported the association between work-family conflict and depressive symptoms in Chinese context. One study on Chinese bank employees found that WFC was positively associated with depressive symptoms [13]. In another study, Chinese professional women’s WFC was positively associated with their depressive symptoms [14]. Another study on Chinese female nurses found that both WFC and FWC contributed to more depressive symptoms [15]. However, little information is known about the association between work-family conflict and depressive symptoms among Chinese employees in the engineering construction industry.

According to the general stress models, a lack of fit at the interface of work and family roles represents a potent stressor that might induce serious mental health problems, such as depressive symptoms [16]. Some studies have distinctively examined the association between WFC or FWC and mental health, because someone may put work before family, while others may value family more importantly than work, thus WFC and FWC may have different impact on employee health outcomes. For example, Frone et al. [17] found FWC was longitudinally related to poor physical health and more depressive symptoms, while WFC was longitudinally related to more heavy alcohol consumption. While other studies found WFC and FWC had similar effects on mental and somatic health [18]. On the whole, both types of conflict go against to employees’ mental health but sometimes with difference in the magnitude of adverse effect. However, we have little information about whether WFC and FWC had the same effect on depressive symptoms in Chinese context. Thus, we focused on Chinese employees in the engineering construction industry and simultaneously examined the effect of the two dimensions of work-family conflict―WFC and FWC on their depressive symptoms and hypothesized the both types of conflict would be positively associated with depressive symptoms.

### 1.3. Work-Family Conflict and Depressive Symptoms: The Role of Organizational Justice Climate

According to the ecological system perspective, individual work-family experience is nested within work and family contexts [19]. Bronfenbrenner’s ecological systems theory suggests that work-family experience reflects the adequacy fit between the individual and his or her environment, which is influenced by process, person, context, and time characteristics [20]. Work-family conflict is often associated with contextual factors in both work and family microsystems, such as work or family pressure [19]. Previous studies also identify many ecological resources such as decision latitude in work, support from coworkers and supervisors, spouse and other family affectual support in work-family interaction process [19]. However, limited studies have considered organizational justice climate and family flexibility as the ecological resources in work-family experience.

Organizational justice describes employees’ subjective perceptions of fairness in their work settings, which is an important contextual factor associated with the interface of work and family [21]. Organizational justice includes four dimensions: procedural justice, distributive justice, interpersonal justice, and informational justice [22]. Procedural justice emphasizes the unbiased decision-making processes in which the interests of everyone should be taken into account. Distributive justice emphasizes the fairness of decision outcomes in which the proportion of investments and outcomes should be consistent for all employees. Interpersonal justice considers the extent to which employees are treated with sincerity and respect by their supervisors. Informational justice refers to adequate and honest explanations about decisions and procedures [22,23].

Organizational justice has been termed as a psychosocial predictor of employees’ psychological and physical health [21,24,25]. Perceived organizational justice could reduce uncertainty and lack of control and in turn reduce the feeling of stress [23]. For example, Ybema and Bos [26] found that distributive justice and procedural justice reduced depressive symptoms in a longitudinal study. Inoue et al. [27] found that low procedural justice and interactional justice were significantly associated with major depressive episodes in Japanese employees in a cross-sectional study. However, these studies have predominantly examined the associations between justice perceptions and depressive symptoms at the individual level.

Recent studies have constructed justice as a collective or group-level concept, which is defined as the justice climate, the degree of fairness perceived by the team as a whole [28,29]. The collective view of justice is based on social interaction, which influences individual perceptions of organizational justice within teams [30]. Building on the social construction of justice perception, justice climate has been defined as group and organizational-level justice perceptions. Workers are expected to be happier when group members believe that they are treated fairly [30]. However, previous studies adopted an individualistic approach to research organizational justice, which fails to take full account of the social context within justice perceptions are shaped [28]. The justice climate is shared through social interactions among individual employees within a work group [28].

According to the social contagion theory, justice climate at the group level would go beyond justice at the individual level to affect individual-level outcomes such as attitudes, behavior, and mental health [30,31]. For example, Spell and Arnold [30] found that distributive and procedural justice climates had a significant influence on employees’ anxiety and depressive symptoms, in addition to the effect of individual perceptions of justice. Lucas et al. [29] found that the justice climate could strengthen the associations between individual-level justice perceptions and personal well-being. In Pecino et al. [32], interpersonal justice climate in a work team was significantly associated with well-being indicators (burnout and engagement) and work outcomes (work family balance and extra-role performance). Employees work in a department with poor justice climate reported more somatic complaints [33].

Organizational justice could not only show a direct positive effect on mental health but also protect employees from the negative effects of workplace risk factors. For example, in a longitudinal study, Gluschkoff et al. [34] found that teachers’ perceptions of organizational justice would weaken the negative effect of workplace violence on their sleep problems. According to the resources conservation model, justice could strengthen individuals’ resources to effectively deal with stress and reduce their vulnerability to stress reactions [21]. In addition, the organizational justice climate could provide a supportive work environment and enhance social interactions among coworkers [28].

In summary, most of previous studies concept organizational justice as a protective factor for employees’ mental health at individual level, but we have limited knowledge about how an organizational justice climate as a work environment variable protects employees from the negative impacts of work-family conflict. Although work causes conflicts with family issues, a justice work environment could respect employees’ contributions and treat employees fairly, employees might feel that work devotion is worth it. Similarly, in a situation in which family issues interfere with work-related responsibilities, a justice work environment could give employees support and make them feel at ease. Thus, work-family conflict would be less stressful in a justice work environment. We hypothesized that an organizational justice climate would protect employees from depressive symptoms induced by work-family conflict.

### 1.4. Work-Family Conflict and Depressive Symptoms: The Role of Family Flexibility

Boundary flexibility is conceptualized as the degree to which an individual can be drawn out of one domain to meet the demands of the other domain, either behaviorally or cognitively [35]. Segmentation and integration are two opposite ends of the segmentation–integration continuum, which characterizes the interface between work and family [36]. When work and family are segmented, the flow between domains is minimized, resulting in reduced domain blurring. On the contrary, when work and family are highly integrated, the flow between domains is maximized, facilitating transitions between domains. According to boundary theory, people develop distinct boundaries around work and family domains [37]. For example, an individual may allow work to flow into the family domain but not allow family to enter the work domain. This highlights the idea that work flexibility and family flexibility are two distinct dimensions of boundary flexibility.

Employees who perceive low boundary flexibility may have difficulties in managing the work-family interface because it interferes with the flow between the two domains. Previous studies have mainly emphasized the importance of work boundary flexibility for reducing work-family conflict. For example, Ferguson et al. [38] found that work boundary flexibility including supervisor instrumental support and organizational segmentation support enhanced family functioning, organizational commitment, and marital satisfaction. However, limited studies have examined the role of family boundary flexibility in work-family conflict due to the lack of formal policies in families [35]. Family flexibility could function as a resource to address stressors that arise from the work-family interface [37]. The family boundary is more flexible; there are more positive spillovers from home to work [35]. In addition, with higher family flexibility, individuals could have more communication with family members about work, which enhances work and family balance [39]. Thus, family flexibility might protect employees from suffering depressive symptoms induced by work-family conflict.

The protective role of organizational justice climate in mental health outcomes may depend on family flexibility. From the ecosystem perspective, work-family experience is a joint function of work and family characteristics. Organizational justice climate and family flexibility can be seen as ecological resources in work and family microsystems, respectively. Communication with family about work and communication with work associates about family are associated with better work and family function, as well as higher work and family satisfaction [39]. Employees with different family roles may have different reactions to organizational support [40]. Those with high family flexibility could flexibly arrange their family tasks and receive sufficient support from family members to ease work-family conflict. However, those with multiple family roles and lack of family flexibility might impose high demands for a family-supportive organization [40]. Thus, employees with low family flexibility are more likely to turn to organizations for help when suffering from work-family conflict, and those with high family flexibility might be less reactive to organizational support. Therefore, we hypothesized that the buffering effect of organizational justice climate in the association between work-family conflict and depressive symptoms would be stronger in conditions of low family flexibility than that in conditions of high family flexibility.

### 1.5. Study Hypotheses

**Figure 1:**
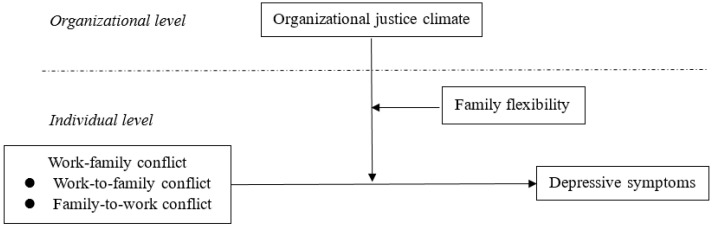
The hypothesized association between work-family conflict and depressive symptoms: The moderating role of organizational justice climate and family flexibility.

According to the work and family interface theory, work-family conflict including both WFC and FWC could be risk factors for employee depressive symptoms. From the ecological perspective, contextual factors in both work and family microsystems are associated with individual work-family experience [19,41]. We identified organizational justice climate and family flexibility as protective factors in work and family settings, respectively, and hypothesized that (see Figure 1):

**Hypothesis** **1:** *Work-family conflict (WFC and FWC) would be positively associated with depressive symptoms*.

**Hypothesis** **2:** *Organizational justice climate (procedural justice, distributive justice, interpersonal justice, and informational justice at the group level) would be negatively associated with depressive symptoms*.

**Hypothesis** **3:** *Organizational justice climate would buffer the association between WFC/FWC and depressive symptoms. That is, compared with employees from groups with low justice climate, the association between WFC/FWC and depressive symptoms would be weaker for employees from groups with high justice climate*.

**Hypothesis** **4:** *The buffering effect of organizational justice climate in the association between WFC/FWC and depressive symptoms would be influenced by family flexibility. Specifically, this buffering effect in conditions of low family flexibility would be stronger than that in conditions of high family flexibility*.

## 2. Materials and Methods

### 2.1. Participants and Procedure

Our participants come from a large state-owned enterprise controlled by the Chinese government. The enterprise is an engineering construction industry and provides engineering, technology, production, sales and other services. As one of the largest Chinese domestic engineering construction companies, this enterprise has many subsidiaries across mainland China. We chose this enterprise as our study sample, because this enterprise is very big and we can get enough participants to test the cross-level interaction. In addition, the subsidiaries in this enterprise have similar organizational structure and management mode, thus the other objective variables in the organization are naturally kept constant, and we can better examine the effect of organizational justice climate at the department level on work-family conflict and depressive symptoms.

As this is a very big enterprise in China and employees in this enterprise face high-intensity work and great work pressure, each subsidiary has an occupational disease center. The survey was completed with the assistance of the occupational disease prevention center at each subsidiary. The staff at these centers were trained in conducting psychological surveys. We explained in detail to them how to guide their employees to complete the survey. We distributed questionnaire at each department’s all-hands meeting and all the employees gave their informed consent for inclusion before participating in this study. The survey took about 30 min. Research assistants collected questionnaires right after their completion. Participants do not necessarily to provide their real name but they could write down their nickname that only they know on the first page of the questionnaire. After 3 months, with the information of enterprise, gender, and nickname, participants could obtain a personal feedback report about their own mental health status. There is no remuneration for their participation in this survey, but they can get a personal feedback as an incentive. In addition, the personal feedback can motivate them to answer the questionnaire seriously and faithfully.

This study was conducted in accordance with the Declaration of Helsinki, and all subjects gave their informed consent for inclusion before participating in this study. As this piece of research in not, by its nature, a clinical experiment, thus it is not necessary to be adjudicated by the Research Ethics of Committee.

This study was conducted in six subsidiaries of this company, and we invited all the employees to participate in this study. Finally, 2365 employees from 103 departments participated in this study. The response rate was 78%. We eliminated departments with fewer than 10 members (a total of 27 departments). As a result, this study included 2184 employees from 76 departments in the data analysis. Each department had an average of 29 employees (standard deviation, SD = 20.10, range = 10–75). The participants had an average age of 42.38 years (SD = 7.61, range = 19–60), and 65% of them were male. The sociodemographic information of participants is displayed in Table 1.

### 2.2. Measures

The questionnaire contained participants’ sociodemographic information, including sex, age, marital status, education, income, and tenure.

*Work-family conflict* was measured with the work-family conflict scale developed by Netemeyer et al. [8]. This scale contained 10 items, with five assessing WFC (e.g., the demands of my work interfere with my home and family life) and five assessing FWC (e.g., the demands of my family or spouse/partner interfere with work-related activities). Participants indicated their responses on a 5-point Likert scale, ranging from 1 (*strongly disagree*) to 5 (*strongly agree*). Higher scores on the two subscales indicated higher levels of WFC and FWC, respectively. In this study, Cronbach’s alpha coefficients for WFC and FWC subscales were 0.88 and 0.83, respectively.

*Organizational justice* was measured with the 20-item justice scale developed by Colquitt [22]. This scale contained four distinct dimensions, including procedural justice (seven items, e.g., have you been able to express your views and feelings during the procedures used to arrive at your outcome?), distributive justice (four items, e.g., does your outcome reflect the effort you have put into your work?), interpersonal justice (four items, e.g., has your supervisor treated you in a polite manner?), and informational justice (five items, e.g., has your supervisor been candid in his/her communications with you?). Responses ranged from 0 (*to a small extent*) to 4 (*to a large extent*). Higher scores on this scale indicated higher justice perception. In this study, Cronbach’s alpha coefficients for procedural, distributive, interpersonal, and informational justice subscales were 0.88, 0.90, 0.87, and 0.89, respectively. Aggregating individual responses about justice perceptions to group level is a common approach to operationalization of climate [28]. Thus, in this study, justice climate was measured by averaging each group member’s justice perceptions within the work group.

*Family flexibility* was measured with the work and family domain boundary flexibility scale developed by Matthews and Barnes-Farrell [37]. The family flexibility–ability subscale consisted of five items (e.g., if the need arose, I could work late without affecting my family and personal responsibilities). Items were rated on a 5-point Likert scale, ranging from 1 (*strongly disagree*) to 5 (*strongly agree*). A higher score on this scale indicated higher family flexibility. In this study, the Cronbach’s alpha coefficient for the family flexibility scale was 0.92.

*Depressive symptoms* were measured with the Patient Health Questionnaire for Depression [3]. This scale has been widely used to measure depressive symptoms. The measurement contained nine items describing depressive symptoms (e.g., little interest or pleasure in doing things). Participants were asked, “Over the last two weeks, how often have you been bothered by any of the following problems?” The responses were indicated by 0 (*not at all*), 1 (*several days*), 2 (*more than half the days*), or 3 (*nearly every day*). Higher scores on the PHQ-9 indicated more depressive symptoms. In this study, the Cronbach’s alpha coefficient for the scale was 0.90.

### 2.3. Statistical Analysis

We used hierarchical regression models to analyze the data, with the individual-level dependent variable (depressive symptoms) regressed on individual-level independent variables (WFC, FWC, and family flexibility) and the group-level independent variable (organizational justice climate). Our hypotheses made predictions about individual-level (Level 1) and group-level (Level 2) variables and their interactions in relation to individual-level depressive symptoms. Before testing these hypotheses, the intraclass correlation (ICC), which describes the proportion of between-group variance to the total variance, was calculated for the dependent variables. We began the analysis by determining intercept and slope terms describing the relationship between predictors and dependent variables in each work group. The intercept and slope terms from this analysis were then used as the dependent variables in between-group analysis. Sociodemographic variables were used as control variables at the individual level, and group size was used as a control variable at group level. We performed the analyses using the Mplus 7.0 program (Los Angeles, CA, USA). To further interpret the moderating effects with figures, high and low levels of moderators were defined as one standard deviation above and below the mean of organizational justice climate and family flexibility, respectively.

## 3. Results

We conducted a confirmatory factor analysis with items loading on its respective variables. The model fit indexes were *χ*^2^ = 8240.437, RMSEA = 0.061, CFI = 0.889, TLI = 0.882, SRMR = 0.060, and the factor loadings of each item ranged from 0.5 to 0.8. We used the bifactor model to test common method variance with all the items loading not only on the construct they belong to, but also on a same latent factor of common method variance. The results showed that the model with latent factor of common method variance cannot be identified, indicating common method variance factor is not reasonable in this data.

The ICC for depressive symptoms was 0.042 (*p* < 0.001), which means 4.2% variance of depressive symptoms existing at the group level. Similarly, the ICCs for procedural justice, distributive justice, interpersonal justice, and informational justice were 0.034, 0.032, 0.035, and 0.032 (all *p* < 0.05), respectively. In a previous study, an ICC of 2% was an acceptable size to justify the use of multilevel analysis [42]. In another study, an ICC of 1.6% was an acceptable size for use of multilevel analysis [43]. Because this study focused on the effect of justice climate at the group level on employee depressive symptoms, it was necessary to use hierarchical linear modeling to address the variance at different levels.

At the individual level, individuals’ perceptions of WFC (*r* = 0.38, *p* < 0.01) and FWC (*r* = 0.44, *p* < 0.01) were positively associated with their depressive symptoms. Individuals’ perceptions of justice were negatively associated with depressive symptoms (*r*_procedural justice_ = −0.27, *r*_distributive justice_ = −0.21, *r*_procedural justice_ = −0.34, *r*_procedural justice_ = −0.29, all *p* < 0.01). Family flexibility was negatively associated with depressive symptoms (*r* = −0.29, *p* < 0.01). At the group level, the collective perceptions of justice were negatively associated with the mean level of depressive symptoms in a certain group (*r*_procedural justice_ = −0.51, *r*_distributive justice_ = −0.39, *r*_procedural justice_ = −0.55, *r*_procedural justice_ = −0.46, all *p* < 0.01). Descriptive statistics and bivariate correlations between the study variables are shown in Table 2.

We conducted hierarchical regression analysis for both WFC and FWC. Table 3 presents the relationship between WFC and depressive symptoms with organizational justice climate (procedural justice in Model 1, distributive justice in Model 2, interpersonal justice in Model 3, informational justice in Model 4) and family flexibility as moderators. The results for FWC and depressive symptoms are presented in Table 4; they are similar to the results of WFC and depressive symptoms in Table 3. 

WFC and FWC were positively associated with depressive symptoms (Table 3, the unstandardized coefficient c = 0.31~0.32, *p* < 0.001; Table 4, c = 0.37~0.38, *p* < 0.001) after controlling for the sociodemographic variables. Thus, Hypothesis 1 was supported. Organizational justice climate was negatively associated with individual-level depressive symptoms (c_procedural justice_ = −0.07, c_distributive justice_ = −0.05, c_interpersonal justice_ = −0.08, c_informational justice_ = −0.06, all *p* < 0.05). Thus, Hypothesis 2 was supported.

**Figure 2:**
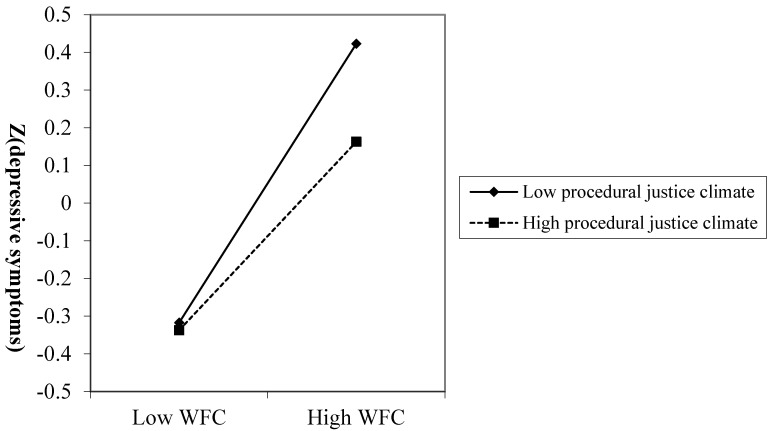
The relationship between work-to-family conflict (WFC) and depressive symptoms was moderated by procedural justice climate. The interaction patterns of WFC × Distributive justice climate, WFC × Interpersonal justice climate, and WFC × Informational justice climate were similar to Figure 2.

In addition, the cross-level interactions of WFC and each dimension of organizational justice climate were significant in predicting depressive symptoms. Decomposing the interaction effect in Figure 2, compared with individuals from groups with low justice climate, the relationship between WFC and depressive symptoms was weaker for individuals from groups with high justice climate. The results for the cross-level interactions of FWC and organizational justice climate were similar to the results of WFC. These results indicated that organizational justice climate could weaken the positive association between work-family conflict and depressive symptoms. Thus, Hypothesis 3 was supported.

**Figure 3:**
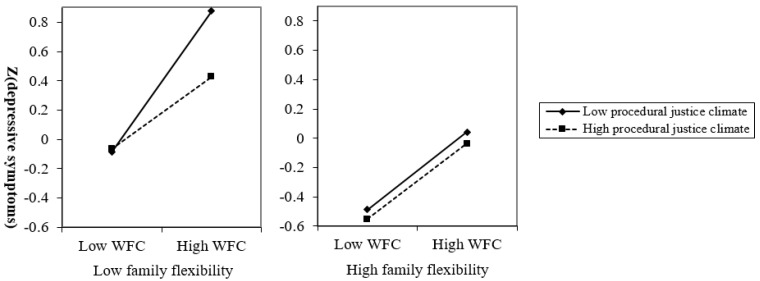
The moderating effect of procedural justice climate in the association between work-to-family conflict (WFC) and depressive symptoms was influenced by family flexibility. The interaction pattern of WFC × Distributive justice climate × Family flexibility was similar to Figure 3.

The three-way interactions of WFC × Procedural justice climate × Family flexibility and WFC × Distributive justice climate × Family flexibility were significant, whereas the three-way interactions of WFC × Interpersonal justice climate × Family flexibility and WFC × Informational justice climate × Family flexibility were not significant in predicting depressive symptoms (Table 3). Decomposing the interaction of WFC × Procedural justice climate × Family flexibility (Figure 3), when family flexibility is low, the interaction of WFC × Procedural justice climate is significant, whereas when family flexibility is high, the interaction of WFC × Procedural justice climate is not significant in predicting depressive symptoms. The interaction pattern of WFC × Distributive justice climate × Family flexibility was similar to the pattern of WFC × Procedural justice climate × Family flexibility. 

The results for FWC and depressive symptoms are presented in Table 4; they are similar to the results of WFC and depressive symptoms in Table 3. These results indicate that the buffering effects of procedural and distributive justice climate in the association between work-family conflict and depressive symptoms became stronger as family flexibility decreased, whereas the buffering effects of interpersonal and informational justice climate were not influenced by family flexibility. Thus, Hypothesis 4 was supported for procedural and distributive justice climate, but not for interpersonal or informational justice climate.

## 4. Discussion

This study examined the association between work-family conflict and depressive symptoms among Chinese employees in the engineering construction industry and further examined how organizational justice climate and family flexibility attenuate the negative effect of work-family conflict using hierarchical regression models. We found that both WFC and FWC—the two dimensions of work-family conflict were positively associated with employees’ depressive symptoms (hypothesis 1). Organizational justice climate was negatively associated with employees’ depressive symptoms (hypothesis 2), and further attenuated the association between work-family conflict and depressive symptoms (hypothesis 3). Moreover, the protective role of organizational justice climate depended on family flexibility. Procedural and distributive justice climate can buffer the negative effect of work-family conflict only for employees with low family flexibility but not for those with high family flexibility (hypothesis 4).

This study provided more evidence about work-family conflict and mental health in a Chinese context. By including WFC and FWC simultaneously, this study found that the conflict between work and family regardless of the directionality could induce employees’ depressive symptoms, which is consistent with most previous findings in Western culture. However, Frone et al. [17] found that FWC was related to more depressive symptoms, but WFC was unrelated to depressive symptoms, according to longitudinal data over 4 years. Our findings were similar with Hao et al. [15], in that both WFC and FWC contributed to more depressive symptoms. As a general emotional state, depressive symptoms would be induced by both WFC and FWC in this study. These results indicate that work and family contexts are fairly important to Chinese employees. Disturbed responsibility in either field would induce employees’ depressive symptoms. Additionally, the relative importance of work and family might change with time. It is reasonable for results among studies conducted in different times and contexts to be inconsistent. Moreover, due to the cross-sectional design of this study, whether WFC and FWC showed the same effect on depressive symptoms in longitudinal studies needs further examination. At any rate, interventions aimed at reducing the conflict between work and family are beneficial to promote Chinese employees’ mental health. China is facing rapid economic development and social changes; Chinese employees’ work-family experience is an important issue. Whether WFC and FWC had the same effect on Chinese employees’ mental health needs further exploration.

This study integrated the organizational justice theory into work-family interface and identified organizational justice climate as an important ecological resource in work environment which would influence employees’ work-family experience. Consistent with the ecological perspective in Grzywacz and Marks [19], work-family experience is a joint function of process, person, work and family context. However, previous studies rarely considered the role of organizational justice climate in work-family experience, and we fill this gap in this study. By taking a collective view of justice, we found that organizational justice climate could protect employees from depressive symptoms. These results indicate that in addition to individual justice perception, justice climate at the group level played an important role in employees’ mental health, which is consistent with previous studies [21,24,25]. In addition, this study found that organizational justice climate weakened the positive association between work-family conflict and depressive symptoms, which indicates that organizational justice climate could protect employees from depressive symptoms induced by WFC and FWC. Previous studies mainly focused on family-supportive organizations to reduce work-family conflict [40,41]. This study indicates that organizational justice climate could be seen as a supportive atmosphere. Employees who perceive their organizations as fair are more responsive to work-family tensions [21]. Aside from suggestions such as flexible working hours mentioned in previous studies [40,41], cultivating a justice atmosphere in an organization is helpful to mitigate the negative effect of work-family conflict.

We also integrated boundary flexibility theory into work-family interface to further understand how organization and family factors influence employees’ work-family experience by simultaneously examining the ecological resources in work and family microsystems [19]. We found that family flexibility could protect employees from the negative outcomes of work-family conflict, which was consistent with previous studies [37,39]. Although family flexibility could not directly weaken the adverse effect of work-family conflict on depressive symptoms, it influenced the moderating effect of organizational justice climate in the association between work-family conflict and depressive symptoms. These results indicate that organizational justice climate and family flexibility could compensate each other to protect employees from depressive symptoms induced by work-family conflict. Specifically, for employees with low family flexibility, organizational justice climate played the dominant role in protecting them from depressive symptoms induced by work-family conflict. For employees with high family flexibility, family flexibility played the dominant role in mitigating the adverse effect of work-family conflict. Family flexibility could reduce time- and strain-based spillover from home to work and work to home [35]. High family flexibility could make transitions between work and family easier, thus mitigating work-family conflict. For example, in the situation in which an employee has to work late, if the employee’s family members could take on family responsibilities for him or her, the work-family conflict could be reduced. However, employees who lack family flexibility are more likely to rely on organizations to mitigate the adverse effects of work-family conflict. Thus, organizations and families should work together to promote employees’ mental health.

Interestingly, we found that the four dimensions of organizational justice climate showed different results in the three-way interaction analysis. The moderation effects of procedural and distributive justice climate on depressive symptoms depended on family flexibility, whereas that of interpersonal and information justice climate on depressive symptoms did not depend on family flexibility. In other words, family flexibility could compensate for the protective role of procedural and distributive justice but not interpersonal and informational justice. Interpersonal justice and informational justice are also called interactional justice, which is a unique dimension of justice [22]. Interactional justice describes whether decision makers treat people with respect and sensitivity and explain the rationale for decisions thoroughly; whereas procedural and distributive justice care more about the interests and outcomes [22,23]. These findings may indicate that the interpersonal and informational aspects of justice play a unique role in the work-family interface, which could not be replaced by family flexibility. The role of each aspect of organizational justice in work-family experience needs further exploration.

### 4.1. Implications

Findings from this study have some implications for reducing the negative consequences of work-family conflict for employees in the engineering construction industry. As the rapid development of construction projects in China, the occupational health of construction workers need more attention. We found their work-family conflict is a serious issue, and interventions at individual, family, and organizational levels should be combined to manage employees’ work-family conflict. First, organizations should cultivate a justice atmosphere in the work setting. For example, organizations could provide employees with opportunities to have a voice and participate in decision-making processes. Supervisors should treat employees with courtesy and respect and act fairly toward all employees. In addition, organizations could create friendly policies, such as flexible hours, to reduce employees’ work-family conflict. Organizations should cultivate a supportive and friendly work environment and provide financial and social support to employees who suffer from family problems. Organizations could provide mental health services to employees who suffer from depressive symptoms. Besides organizational justice climate, other organizational factors such as organizational structure and work arrangement in alleviating work-family conflict can be considered in future studies. Second, employees’ work-family conflict and depressive symptoms could be reduced by enhancing their family flexibility. Spouses should promote mutual support and understanding and share family responsibilities, such as housework and taking care of children. These findings hint that family flexibility is an essential condition for construction workers. Future studies may take other family characteristics such as spouse’s characteristics into work-family conflict research. Lastly, organizations and family members could provide support to enhance employees’ ability to manage and flex the boundaries between work and family. In China, now employees’ mental health is not compulsively monitored by an occupational doctor. The provision of mental health service is not mandatory in Chinese legislation but is up to the enterprise. Thus, enterprises should consider personalized interventions to reduce employees’ work-family conflict and depressive symptoms. For example, enterprises should pay more attention to employees who lack family flexibility.

### 4.2. Limitations

We have to acknowledge some limitations in this study. First, although we theoretically identified work-family conflict as the antecedent of depressive symptoms, we cannot infer a causal relationship between work-family conflict and depressive symptoms with this cross-sectional design, which can be tested in future longitudinal or experimental studies. Second, the variables in this study were assessed with self-reported data, but fortunately, we statistically tested the common method bias to be nonsignificant. Future studies may consider multiple data source. Third, this study was conducted in Chinese engineering construction industry. Whether the results can be generalized to other enterprises or other countries requires further examination.

## 5. Conclusions

Employees’ both WFC and FWC are positively associated with their depressive symptoms. Organizational justice climate could weaken the effect of work-family conflict on depressive symptoms. The buffering effects of organizational justice climate in the association between work-family conflict and depressive symptoms only exist for employees with low family flexibility but not for those with high family flexibility. Interventions at individual, family, and organizational levels should be combined to reduce employees’ work-family conflict and improve their mental health.

## Figures and Tables

**Table 1:** Sociodemographic characteristics of the participants.

Characteristics	Mean ± SD (Range) *n* (%)
Age	42.38 ± 7.61 (19–60)
Tenure	21.81 ± 8.88 (0.5–42)
Gender	
Male	1419 (65.0%)
Female	765 (35.0%)
Marital status	
With a spouse	1962 (89.8%)
Single (divorced/widowed/unmarried)	222 (10.2%)
Education	
Secondary education or below	838 (38.4%)
Junior college	655 (30.0%)
Tertiary education	691 (31.6%)
Monthly income (RMB)	
<3000	223 (10.2%)
3000–4000	1008 (46.2%)
4001–5000	608 (27.8%)
>5000	345 (15.8%)

Note. 1 USD = 6.5 RMB (Renminbi). SD = standard deviation.

**Table 2:** Descriptive statistics and bivariate correlations of the study variables at individual level and group level (*n* = 2184).

Variable	Mean ± SD	1	2	3	4	5	6	7	8
**Individual level**									
1. Work-to-family conflict	13.75 ± 4.70	--	0.57 **	−0.23 **	−0.13 **	−0.13 **	−0.20 **	−0.15 **	0.38 **
2. Family-to-work conflict	10.00 ± 3.80		--	−0.29 **	−0.06 **	−0.02	−0.22 **	−0.12 **	0.44 **
3. Family flexibility	18.40 ± 4.30			--	0.28 **	0.22 **	0.34 **	0.30 **	−0.29 **
4. Procedural justice	13.65 ± 5.46				--	0.69 **	0.72 **	0.83 **	−0.27 **
5. Distributive justice	7.04 ± 3.64					--	0.55 **	0.59 **	−0.21 **
6. Interpersonal justice	9.72 ± 3.20						--	0.85 **	−0.34 **
7. Informational justice	11.10 ± 4.10							--	−0.29 **
8. Depressive symptoms	15.30 ± 5.22								--
**Group level**									
4. Procedural justice climate	13.86 ± 1.68				--	0.74 **	0.80 **	0.88 **	−0.51 **
5. Distributive justice climate	7.12 ± 1.08					--	0.54 **	0.60 **	−0.39 **
6. Interpersonal justice climate	9.88 ± 0.92						--	0.90 **	−0.55 **
7. Informational justice climate	11.25 ± 1.20							--	−0.46 **
8. Depressive symptoms	15.12 ± 1.62								--

Note. ** *p* < 0.01.

**Table 3:** Work-to-family conflict and depressive symptoms: Cross-level interactions of organizational justice climate and family flexibility.

Level and Variable	Model 1	Model 2	Model 3	Model 4
Level-1 (*n* = 2184 individuals)				
Gender	0.07	0.07	0.07	0.07
Age	0.00	0.00	0.00	0.00
Education	−0.13 **	−0.13 **	−0.13 **	−0.13 **
Marital status	−0.11	−0.11	−0.11	−0.11
Income	−0.14 **	−0.14 **	−0.15 **	−0.14 **
Children	0.03	0.02	0.02	0.00
WFC	0.31 ***	0.32 ***	0.31 ***	0.31 ***
FF	−0.16 ***	−0.17 ***	−0.17 ***	−0.17 ***
WFC × FF	0.00	0.00	0.00	0.00
Level-2 (*n* = 76 departments)				
Group size	0.00	0.00	0.00	0.00
Procedural justice climate	−0.07 ***			
Distributive justice climate		−0.05 **		
Interpersonal justice climate			−0.08 ***	
Informational justice climate				−0.05 *
Cross-level interactions				
WFC × Procedural justice climate	−0.06 ***			
FF × Procedural justice climate	0.03			
WFC × Procedural justice climate × FF	0.01 **			
WFC × Distributive justice climate		−0.07 **		
FF × Distributive justice climate		0.01		
WFC × Distributive justice climate × FF		0.01 **		
WFC × Interpersonal justice climate			−0.07 ***	
FF × Interpersonal justice climate			0.03	
WFC × Interpersonal justice climate × FF			0.00	
WFC × Informational justice climate				−0.06 **
FF × Informational justice climate				0.01
WFC × Informational justice climate × FF				0.00

Note. The table shows unstandardized coefficients. WFC = Work-to-family conflict, FF = Family flexibility, * *p* < 0.05, ** *p* < 0.01, *** *p* < 0.001.

**Table 4:** Family-to-work conflict and depressive symptoms: Cross-level interactions of organizational justice climate and family flexibility.

Level and Variable	Model 1	Model 2	Model 3	Model 4
Level-1 (*n* = 2184 individuals)				
Gender	0.04	0.04	0.04	0.04
Age	0.00	0.00	0.00	0.00
Education	−0.12 **	−0.12 **	−0.12 **	−0.12 **
Marital status	−0.14	−0.14	−0.14	−0.14
Income	−0.07	−0.07	−0.07	−0.07
Children	0.06	0.05	0.06	0.05
FWC	0.37 ***	0.38 ***	0.37 ***	0.37 ***
FF	−0.13 ***	−0.14 ***	−0.13 ***	−0.13 ***
FWC × FF	0.00	−0.01	0.00	0.00
Level-2 (*n* = 76 departments)				
Group size	0.00	0.00	0.00	0.00
Procedural justice climate	−0.11 ***			
Distributive justice climate		−0.09 ***		
Interpersonal justice climate			−0.10 ***	
Informational justice climate				−0.09 ***
Cross-level interactions				
FWC × Procedural justice climate	−0.06 **			
FF × Procedural justice climate	0.06			
FWC × Procedural justice climate × FF	0.01 *			
FWC × Distributive justice climate		−0.06 *		
FF × Distributive justice climate		0.03		
FWC × Distributive justice climate × FF		0.01 **		
FWC × Interpersonal justice climate			−0.06 *	
FF × Interpersonal justice climate			0.05	
FWC × Interpersonal justice climate × FF			0.00	
FWC × Informational justice climate				−0.05 *
FF × Informational justice climate				0.04
FWC × Informational justice climate × FF				0.00

Note. The table shows unstandardized coefficients. FWC = Family-to-work conflict, FF = Family flexibility, * *p* < 0.05, ** *p* < 0.01, *** *p* < 0.001.
